# Young Registered Nurses' Intention to Leave the Profession and Professional Turnover in Early Career: A Qualitative Case Study

**DOI:** 10.1155/2013/916061

**Published:** 2013-08-20

**Authors:** Mervi Flinkman, Ulpukka Isopahkala-Bouret, Sanna Salanterä

**Affiliations:** ^1^Department of Nursing Science, University of Turku, 20014 Turku, Finland; ^2^Faculty of Behavioural Sciences, University of Helsinki, P.O. Box 9, 00014 Helsinki, Finland; ^3^Hospital District of Southwest Finland, Bureau of Administration, Turku University Hospital, P.O. Box 52, 20521 Turku, Finland

## Abstract

In a time of global nursing shortages an alarming number of young registered nurses have expressed a willingness to leave the profession. In this qualitative case study we investigate in depth why young nurses leave nursing profession and reeducate themselves for a new career. The study is based on longitudinal interviews of three young registered nurses in Finland. These nurses were first interviewed between December 2006 and May 2007, when they were 29–32 years old and having an intention to leave the profession. The second interview took place four years later, from January 2011 to March 2011 when all of them had made the transition to a new career. Data were analyzed in two stages. In the first stage, comprehensive career story narratives were formed on the basis of the interviews. In the second stage, emerging themes in these stories were compared, contrasted, and interpreted in the context of the overall career histories. Nursing as a second career choice and demanding work content as well as poor practice environment and the inability to identify with the stereotypical images of nurses were main themes that emerged from these career stories. The results of this interpretative qualitative study reflect a shift toward insights into understanding professional turnover as a complex and long-lasting process.

## 1. Introduction

Nurses are the largest group of professionals within the global health care system, with a total of 19.3 million nursing and midwifery personnel in the world [1]. The current and growing shortage of registered nurses (RNs) in health care systems is thus a global concern [2, 3]. In fact, the European Commission has estimated that there will be a shortage of 590,000 nurses by the year 2020 [4]. In the United States, employment of RNs is expected to grow faster than the expected average for all occupations [5]. Most countries within the Organization for Economic Cooperation and Development (OECD) have reported a nursing shortage [6], which is predicted to get worse because the current nursing population is aging [7]. This shortage of RNs influences the delivery of health care and negatively affects patient outcomes; an insufficient nurse staffing level is associated with negative patient outcomes [8, 9] and decreased nurse job satisfaction [10]. 

At the same time of this global nursing shortage, many nurses are considering leaving their job, profession or are out of the nursing workforce. According to Flinkman et al. [11] literature review, nurses' intention to leave the profession varied from 4% up to 54% across the studies internationally. In a NEXT (nurses early exit) study, conducted in ten European countries (*N* = 30,330), 13% of nurses had thought about leaving the profession frequently [12]. According to an RN4CAST (nurse forecasting in Europe) study (*N* = 33,659 nurses/Europe; 27,509 nurses/United States), the proportion of nurses planning to leave their current job ranged from 49% (Finland, Greece) to 14% (United States) [13]. In European sample of this study, every tenth (9%) of nurses was having intention to leave the profession [14]. Furthermore, in Salminen [15] study, nearly half (37%, *N* = 343) of young RNs (under 35 years) working in hospitals have reported frequent intention to leave the profession in Finland. In Sweden, 10–20% of new graduates have considered leaving the profession [16]. In the United States, it was reported that the percentage of RNs intending to leave nursing within 3 years was low, at 3%. Yet, of the total licensed RN population in 2008, it was estimated that more than 15% (*n* = 466,564) were not actually employed within nursing [17]. 

Turnover intention appears to be a multistage process consisting of psychological, cognitive, and behavioral components [18] and has been found to predict the actual decision to leave the profession [12, 19, 20]. According to a study by Hasselhorn et al. [12], the majority of leavers began the process with serious consideration in the final year preceding leaving, and the actual decision to leave was then made within the 6 months prior to determination. In Carless and Arnup's [21] study, one year prior to changing careers, actual career changers were actively looking for a new career and had a high intention to leave their current job. Furthermore, another study revealed that nurses left nursing within 6 months of their decision to leave [22]. The final decision to leave the profession is likely to be the result of an individual reflection process [21, 23] with multiple underlying causes [11, 12].

From the society's and healthcare's points of view, professional turnover is a more significant form of work transitions than organizational turnover [24, 25]. Those RNs, who are leaving the profession, are reducing the total number of nurses in the manpower, which has an impact on the present nursing shortage [2] and is leading to a permanent loss of productivity [18]. Nurses leaving the profession take their tacit knowledge, experience, and contribution from the organizations and also from the nursing workforce [11]. The financial investments used on nurse's education, orientation, and continuing education are lost. Moreover, nurse turnover is also costly to organizations: first, because it results in the direct and indirect costs of filling the positions, and second, because of the loss of organizational productivity and knowledge [26]. 

The youngest generation of nurses are the most willing to leave the job and the nursing profession [12, 15, 16, 27, 28]. Kovner and Djukic [29] have reported that in the United States more RN graduates left their first nursing job (26%) than nursing profession (2%) during the first two years in career. According to NEXT study [12], in most European countries the intent to leave the profession was highest in the age groups between 25 and 35 years of age. In Robinson et al. [30] study, diploma-qualified nurses' movement into other activities was highest around the age of 28 and declined thereafter. According to Barron and West [31] study, rate of leaving the nursing profession for a better job was highest at the age of 32. However, conflicting findings also exist. According to an RN4CAST-study, older nurses were having higher intention to leave the profession than younger nurses [14].

Several factors are related to young RNs' intentions to leave the profession, including an imbalance of effort and reward, high psychological demands, and higher job strain, which all influence young nurses' intention to resign from their nursing careers [32]. According to a Swedish study of Rudman et al. [16], graduates of a younger age are more vulnerable to early career burnout, which is associated with the intention to leave the profession. In a study by Flinkman et al. [33], both the survey questionnaire and the open-ended questions showed that young nurses' intentions to leave the profession were connected with the highly demanding work, burnout and dissatisfaction with salary levels. 

Experiences of resent graduates have been widely examined in the nursing research [34–36]. However, less research is available concerning graduate nurses' intention to leave the profession. In a study by Scott et al. [37], graduates (mean age 29) who did not like being a nurse reported a higher desire to leave the profession. In a study by Lavoie-Tremblay et al. [38], those young graduates (24 or younger) who reported having poor work environment, were more likely to state they would leave the profession. 

Most of the earlier turnover research is concerned with nurses leaving the job or the organization [27]; less research is conducted on what motivates nurses to leave their profession [11, 25]. This is understandable, because nurse turnover is expensive and organizations are usually more concerned with their own nursing manpower than workforce in a whole [11]. Studies concerning nurses' turnover and their intent to leave the profession have been mainly executed using quantitative methods and survey questionnaires [11, 27, 39], revealing the need to explore this phenomenon using qualitative methods and to produce studies with more in-depth understanding. Accordingly, qualitative research and research from generational perspective has been recommended in order to get a more comprehensive picture of turnover [27, 40].

Knowing that young nurses' transition from a student to nursing career is a demanding and stressful process and thus could lead to nurse turnover from the profession [37, 38], it is important to explore views and perceptions regarding early career experiences from the young nurses point of view. Therefore, the aim of this study was to gain a deeper understanding of young nurses' career transition processes, as experienced by young registered nurse, aged between 29 and 32 years. Young registered nurse (RN) in this study refers to a person who has completed Bachelor of Health Care degree in the University of Applied Sciences and whose intention to leave the profession has started before the age of 30 years.

## 2. Material and Methods

### 2.1. Design

This inquiry has been designed as a longitudinal qualitative case study using interpretive narrative method. A “case” is being defined as a longitudinal career story interpreted by a young registered nurse.

### 2.2. Theoretical Framework

This is a qualitative case study [41], in which an interpretative narrative approach [42, 43] is adopted. Qualitative case study has been defined as a research methodology grounded in an interpretive, constructivist paradigm [41]. Case studies often contain narratives that approach the complexities and contradictions of real life [44] and can be used for exploring, describing, and understanding a phenomenon in its context [44, 45]. An intensive investigation of cases can provide contextual knowledge of real-life phenomena that would be unobtainable with a population-based approach [45]. Case-centred research can add knowledge of how people experience career transitions and construct new career identities [46]. 

According to Sharp [47] case-centred approaches are an excellent and relevant way of doing nursing research, because nursing practice is centred on cases (patients and in broader sense organizations and social context). Even though “nurses have not fully embraced case study as a comprehensive approach for research” [45, page 103], case studies can be seen as an option for many nursing studies, because it allows to use various sources of evidence and it has a potential to uncover multiple realities [48]. 

Both the interview material and data analysis method in this study can be defined as narrative. The underlying assumption of narrative inquiry is the idea that individuals make sense of their world by telling stories [49]. Narrative approach does not assume objectivity, but rather it privileges positionality, pluralism, relativism, and subjectivity [50, 51]. There is an underlying assumption in a narrative approach that “there is neither a single, absolute truth in human reality nor one correct reading or interpretation of a text” [50, page 2]. Narrative turn is a part of a paradigmatic shift from realism toward constructivism in science, which then further refers to fundamental changes in beliefs concerning reality and knowledge [52]. 

Events in a narrative are selected, organized, connected, and evaluated in accordance with the audience to which the narrative is told (e.g., the interviewer) [53]. Interpretation is inevitable because stories are representations, and a researcher does not have direct access to another's experiences [51]. Stories are usually composed and received in context, telling much about the society, culture, and the social reality the teller is living in [54]. Stories are a way for a person to make sense of disruptive events in their lives or when there has been a breach between the ideal and real, self and society [51].

Cochran [55] suggests that narrative study is a paradigm in career research, because the topic of the career is not ultimately focused on the distinct parts of the story but instead on how the different elements of a professional history are interrelated and brought together. Transitions from one profession to another involve always narrative reflection [56]. By narrating their professional history and transitions, people try to find a meaningful connection between their past and new careers [46]. A successful transition to a new workplace or occupation could be considered as completed once a person is able to resolve the conflicts and contradictions in his or her career narrative [57]. 

### 2.3. Recruitment Procedure and the Participants

The selection of participants was purposeful [53], as we were seeking “key informants” or “critical cases” that were knowledgeable about the phenomenon of interest [44]. In the year 2006, we invited young nurses to write the narrative stories of their nursing careers and why they had an intention to leave nursing. We advertised in *Tehy*—the magazine of the Union of Health and Social Care Professionals—and in an open discussion forum for nurses via the Internet (http://www.hoitajat.net/). To be eligible for the study, we required the participants to

- be a registered nurse,
- have work experience in nursing,
- have an intention to leave a nursing profession that had started before the age of 30,
- have a willingness to articulate, and reflect upon, their experiences in their nursing career.

We requested that they add their contact information if they wanted participate the interviews concerning the intention of leaving the nursing profession. 

Three young female registered nurses were willing to tell their story to the interviewer and met the inclusion criteria. We decided to accomplish longitudinal career story interviews with these three nurses in order to get an in-depth understanding of the phenomenon of interest. We considered that three young nurses was a sufficient number, because in case study method sample sizes are nonrepresentative [47] and narrative interview data usually accumulate with a large quantity of rich data [51]. The participants were 29–32 years old during the course of the first interview. The nurses were living in different parts of Finland, one in a large city and the remaining two in smaller cities. They all had degrees of Bachelor of Health Care. Nursing education in Finland is arranged in universities of applied sciences, and study consists of 210 credit points. 

### 2.4. Conducting the Interviews

Young nurses were interviewed twice. The first author conducted all of the interviews: a female researcher who was at the time in her 30s, a registered nurse, and a PhD student in nursing science. The process of gathering the initial data was conducted between December 2006 and May 2007. In the beginning of the first interview the interviewees were asked to draw—on A3-sized paper—their pathway as a nurse and put into this drawing the significant events in, and experiences of, their nursing career. Narratives of personal experiences or life stories are often told in response to open-ended questions, which do not limit or specify the type and the form of the narrative [58]. In the first interview, an open question was posed: *“I would like you to tell me, freely and with your own words, your story as a nurse. You can start your story at any point of your life that you have felt to be significant for you becoming a nurse. You can end your story at any point in your life that you want.” *After this open-ended question, the interviewer acted as an active listener and avoided interrupting the participant's narrative. 

When the complete story had been obtained, some detailed relevant questions were presented to encourage further elaboration on the narrative (e.g., “*could you tell more about why you applied to nursing school?*”). Further questioning helps participants to recall details, turning points, and other meaningful events in their narrative story [53]. In the last phase of the interview, the interviewer asked the participant whether she wanted to say anything else; the interviews ended when the participant did not want to add anything further.

The second interviews took place four years later, between January and March 2011, when all of these young nurses had made the transition to a new career. In the beginning of the second interview, the participants were asked to continue their earlier career-pathway drawing and were asked to continue their story of being a nurse from any point in their life that they chose.

The lengths of the interviews varied from 113 minutes (longest first interview) to 25 minutes (shortest second interview). One nurse chose to be interviewed at her workplace, and the other participants were interviewed in their homes. Interviews were recorded on a digital recorder and were transcribed verbatim by the first author, because analysis and interpretation of such data depends on the adequacy of a carefully prepared manuscript [59]. 

### 2.5. Data Analysis

Narrative data guided the data analysis method we chose for this inquiry. Data were analyzed in two stages. Firstly, we followed the holistic-content method developed by Lieblich et al. [50], where the life story of a person is taken as a whole; researchers studying life histories and case studies also use this approach. After the first readings of the data, the first author formed three career story narratives on the basis of the interviews, career-pathway drawings and written stories. In these narratives, the first author selected and organized the separate events from the nurses' careers into complete narratives with a chronological order. Polkinghorne [42] describes this kind of method as narrative analysis, where the researcher configures a new narrative on the basis of the narrative material. These career stories were then reviewed and discussed with the coauthors.

We found in the preliminary readings that while these narratives were unique, certain main themes also appeared to be reflected both within and between these stories. A thematic approach to narratives is useful when trying to find common thematic elements across research participants and the events they report [53]. As Sandelowski and Leeman [60, page 1407] conclude, “The identification of themes is foundational to qualitative research of all kinds”. The first author followed these particular themes throughout the stories and compared, contrasted, and interpreted these themes in the context of the overall career histories. These emerging interpretations and themes were discussed, reviewed, and refined with the coauthors. The results of the analysis are reported in accordance with these themes. By presenting cases and themes simultaneously it was possible to reach a more holistic and multidimensional perspective to these stories.

### 2.6. Trustworthiness

To evaluate the validity of narrative studies, Riessman [43] argues that it is trustworthiness, rather than truth, which should be evaluated in narrative studies. We established the trustworthiness for this study in four ways. First, our data analysis is described in detail, and direct citations from the narratives are provided in relation to each story to reveal the basis from which the analysis was conducted. According to Polkinghorne [61, page 6], “Readers should be able to follow the presented evidence and argument enough to make their own judgment as to the relative validity of the claim.” Second, we used data triangulation (narrative interviews, career-pathway drawings, and written stories) to enhance the trustworthiness of the analysis. Third, the narrative stories and themes were reviewed in research seminars in a doctoral school of nursing science and in a narrative summer school to ensure that the chosen themes resonated with the narrative stories. Fourth, the accuracy was reviewed by sending the final paper draft to the participants, to allow them to verify the authenticity of the data and ensure that they agreed with the interpretations. As the original transcripts were in Finnish, the citations used in this paper have been translated into English by a professional translator in order to reproduce the citations as accurately as possible and maintain the authenticity of the participant's responses. 

### 2.7. Ethical Considerations

We followed the ethical guidelines described by the Finnish Advisory Board on Research Integrity [62]. The participants gave their oral and written informed consent to participation. We informed them that they had the right to withdraw at any stage, and that the collected data would be treated with confidentiality. We removed the following details from the career stories: places of residence, names of the health care organization, names of family members, and names of colleagues. We also changed some minor details in these career stories, which we considered to be insignificant, in order to protect participants' identity. Furthermore, we used pseudonyms to protect participant anonymity. Because nurses participated voluntarily and in their leisure time, no additional ethical committee approval was needed.

## 3. Results and Discussion

### 3.1. Starting the Stories: Introducing the Career Story Narrators

At the time of the first interview, Anna (a pseudonym) was a young woman at a crossroad in her nursing career. She showed a strong intention for a career change, yet at the same time she was tired and distressed because she was not sure how such a career change would be possible. Anna was very analytical and told her story in an animated way. Her narrative was detailed, complex, and often included cynical humor and sarcasm; she also often vocalized direct quotations from other people's speech to make parts of her narrative more dramatic. Anna used a great amount of time in the first interview analyzing what she wanted for her future career and what she might be capable of doing if she were to leave nursing work. She had talked about this career change with her relatives and friends and had thought deeply about the advantages and disadvantages of leaving the nursing profession. 

Betty (a pseudonym) already had a new university education at the time of her first interview. She told her story in a good-humored way, often laughing. During her story, Betty mentioned frequently that her nursing job had mostly been “nice” and the patients were also mostly “nice”. There were some more dramatic stories—for example, an unsuccessful resuscitation of a small baby—but mainly her nursing career story was positive and optimistic. She had, however, stayed in her nursing career for quite a short time.

Cecilia (a pseudonym) was very calm and analytical when telling her story. Her narratives were well structured, and she used a flowing narrative style to tell her stories. Cecilia had mainly enjoyed nursing work and taking care of patients. She had a strong sense of treating other people in a fair and just way. During her time at the nursing school confidential tasks were already being given to Cecilia, and teachers were saying that she would become a good teacher. She had already begun to be interested in management when she was in the nursing school and was willing to develop herself professionally from the beginning of her nursing career. 

### 3.2. Theme One: Nursing as a Second Career Choice

When Anna was in both grammar school and high school, she had no intention of becoming a nurse. She considered many careers, for example, a veterinarian or journalist, but was not sure, or at least confident, of what she wanted to do in her forthcoming career. After high school she applied to university to read nonnursing discipline but was not accepted. She was at that time in practical training in a hospital and liked the work, despite the fact that it was not exactly the work she was considering as a career choice. Not knowing what else to do, she however applied to a nursing school and was accepted. Anna related that she did not know anything about nursing when she was applying to nursing school, but she was confident that she was never going to work as a bedside nurse. In the admission test she achieved a very high overall score but a low motivation score. In her retrospective analysis she reflected that the test had probably quite accurately measured the reality of the situation: she was not especially eager to get into the nursing school. At that time her mother and friends had said that a career in nursing might not be right for Anna's personality.

*Well, it was some sort of drifting, that I had never any kind of calling. And then I thought that I really did not know where to apply, so then it was the nursing school, because perhaps nursing was something kind of similar to psychology, although I did not really want to admit to myself that it wasn't really like that.*

Betty was also not interested in a nursing career when she was in high school. After high school Betty applied to university but, disappointingly for her, was not accepted. She was doing voluntary work during a “gap” year and through this became interested in nursing work. She decided to apply for a nursing education because she thought that nursing could be a stepping stone for working in developing countries or in becoming a physician. At the very beginning of her nursing studies she had already started to hesitate: was she choosing the right career? In the beginning of her story she recalled

*I do not even know how to say why I ended up becoming a nurse, but I feel that I have sort of drifted or something.*

Cecilia too believed that she became a nurse through serendipity, and a nursing career had not been a childhood dream for her either. After high school Cecilia decided to move abroad and to start university studies there. Before leaving, Cecilia was working for a few months in a hospital and started to consider what it would be like to be a nurse. Coincidentally she noticed an advertisement for the nursing studies in a newspaper, decided to apply, and was accepted. One of her relatives had worked in a hospital when Cecilia was a small child. She thought that there was a certain attraction to nursing work, although at the same time the hospital was quite a frightening place, with white working clothes and with peculiar smells. She recalled

*But a nursing career was never a dream job for me. It's not as if I had thought that I would like to work in a hospital. Maybe this has been more like destiny that I have gone there. *

Even though these young nurses did not have nursing as a first career option, they all graduated from nursing school with good grades. They also all received positive feedback from the school and from clinical practices, and their memories from the nursing school were mainly positive in their narratives. 

### 3.3. Theme Two: Demanding Work Content and Poor Practice Environment

Since the beginning of her nursing career, Anna had been working in a ward where many patient were alcoholic, drug abusers, or elderly patients with no hope of recovery. She said that she did not like the routine tasks related to patient hygiene and housekeeping within the ward. Anna did not like working close to the patients; she highlighted nursing tasks that were menial, unpleasant, and intimate. Perhaps because of the work-related burnout, she recalled

*I'm just so irritated by our patients to be honest. When we think of the picture that the so-called “normal” people have of a hospital, that there nurses take care of car-crash patients and make them healthy and heal them and all kinds of things. And the reality is some are intoxicated, some alcoholic, and then there are old people with many diseases whose nursing feels more like torturing them. *

Betty also had to take on a great deal of responsibility as a graduate nurse: in some wards she was the only RN during a shift, as all the others were nursing aids. There was insufficient staffing level, and patient care was demanding. When she was employed in her last summer job as a nurse she thought that the nurses where she was working had an increased workload, and that they were extremely busy. She was no longer enjoying nursing work, and she felt that being productive was seen as more important than taking good care of the patients. She told a story about one nightshift when the workload was almost overwhelming

*That hospital, it was a place like, it was really something like eight people in the same room and some of those people were screaming all night long. That's also when I thought that this is not making any sense! And there's two of us nurses and over forty patients on the wards. That was totally insane.*

Cecilia liked nursing work related to the patients and their care and enjoyed both the atmosphere of the workplace and her coworkers. The nursing job was demanding but also rewarding. At the same time as relating how much she liked her job, Cecilia also verbalized feelings of guilt and of being burdened because she thought that she was unable to provide the level of care that she considered adequate. She told a story of her last summer in the hospital when there were many inexperienced doctors on the ward. She had subsequently thought that she had to supervise and look after those doctors. She recalls

*We had had some really difficult patient cases and that summer for some reason there happened to be some really, really inexperienced doctors at the beginning of their careers, candidates of medical science, and all the time you had to be kind of mentoring their actions. It felt like you had to watch out all the time, and that besides your own work you kind of had to also take care of how everything else runs there. *

There was a strong hierarchy in the hospitals, and the lowest in the hierarchy were nursing students—in some workplaces they were not even permitted to go into the coffee room—yet graduate nurses and new nurses in the ward were also low down. Betty noticed during her nursing education that medical students were even lower in the hierarchy than nursing students; after this realisation she decided against pursuing a medical career. Strong hierarchy was a disappointment for these young nurses; however, they felt that they could not change the situation. 

During her interviews, Cecilia often recounted her experiences of poor management in the hospital. For example, she had supervised students, often using her own leisure time to do so, but these efforts were not compensated; furthermore, she had lacked feedback about what she was good at, and what she needed to do in order to develop. She had wanted to participate in further education, but as a temporary worker she was not offered this possibility. Cecilia felt that managers in the hospital did not actually understand how demanding the nurses' work was, and that they had very unrealistic proposals about how to develop patient care. She also confronted ethical problems in the ward; she felt that nurses in the ward were not able to provide humane and proper care. She recalled

*And then, like I told you, my husband came to pick me up from work and then I started to cry that I cannot take this anymore, this is terrible, I cannot. I cannot, I feel like nobody takes good care of these patients.*

Anna felt that nursing would not provide her with intellectual challenges after a few years of work, and that nurses had no career development possibilities. Similarly, Betty had sought career development opportunities but was not able to see the potential for pursuing them in her nursing career; she had hoped that nurses would have some kind of career ladder, to allow for such progress. Thus, this lack of professional development was a common concern, as expressed by Betty

*I find it kind of terrible to think that you graduate as a nurse and then you will be a nurse for the rest of your life. That maybe you move from one ward to another ward or from place to place but the work is always the same everywhere.*

All of these nurses were dissatisfied with their salaries. Anna thought that even a much higher salary would not compensate for work that was otherwise unsatisfactory. Betty said that the salary was too low when compared to the responsibilities of nursing work; she was getting a better salary in her new job outside nursing, even though there she was not able to “kill anyone.” She also thought that higher salaries would improve the image of nursing. 

Anna, Betty, and Cecilia all did shift work during their nursing career. Combining private life with shift work was demanding for them, because they found planning their lives around it challenging. Recovering from night shifts was also difficult, because it was hard to be awake at nights and sleep during the day. Betty, furthermore, said that if she did not get enough sleep, she was prone to suffer from migraines. 

All of these nurses were working under fixed-term contracts at the beginning of their career, because during the economic depression, graduate nurses were mainly offered only fixed-term employments. Anna felt that the charge-nurse in the ward was using fixed-term contracts as a way to exert power. This power relation changed when there started to be a shortage of nurses in the ward, which provided Anna with a bargaining opportunity. After telling the ward sister that she was looking for a new job, she was offered longer fixed-term contracts. Anna finally secured a permanent work contract after a few years. She thought that getting a permanent job was a good thing for gaining control over one's own life, although at the same time she also thought that she was then somewhat “trapped.”

*There were these fixed-term contracts which could be from two weeks to three months or then for six months. They couldn't give me their word that there would be longer periods which made me feel really insecure when I thought about my future.*

Betty did not work in nursing for long and only with temporary work contracts. While studying for a new career she worked for short periods in numerous hospitals. In some places there was no proper orientation for the job, and there was no support for a recently graduated, inexperienced nurse. Cecilia also started with temporary work contracts, working in many different hospitals. She had the possibility to apply for a permanent contract but never did so; she did not want a permanent job because she was sure that she was not going to work for the next forty years as a nurse.

### 3.4. Theme Three: The Inability to Identify with the Stereotypical Images of Nurses

Within the narratives, all these young nurses described what kind of people nurses are: nurturing, altruistic, and willing to serve. They did not, however, place themselves within these expected images of nurses. In their stories, these young nurses narrate nursing as a profession, not as a vocational calling. They construct themselves as talented and ambitious and striving for career advancement. Throughout Anna' story, she described herself as intellectual, creative, and systematic, but that she was not able to use any of these qualities in nursing. She thought that many other nurses were hoping to change career but found it impossible because of the demands of their families. Her boyfriend concurred, as he had said that she was too bright and talented for a nursing work

*My husband used to tell me “well with that mind, with those talents, with that brain, it is obvious you're not going to enjoy it there”, that he has always been saying to me that you're in the wrong profession.*

Betty also related that she had not been a nurturer when she was a child. When Betty applied to the nursing school, her parents said that she was too ambitious for a nursing career, and that she would not stay in nursing for long. However, Betty was a strong-minded woman and decided at that time to continue, despite her parents' worries. After graduation, Cecilia worked on a ward taking care of elderly patients. She was afraid that she was going to be “stuck” in that ward, where she was not capable of using her knowledge and talents as a nurse. Cecilia also produced stereotypical images of nurses in her stories: she described that nurses had a role within the hospital of the physicians' handmaids. She thought that nurses had a lower professional status in the hospital than physicians and that a nurse's role was not autonomous.

*Nurses are physicians' handmaids in the hospital. So developing your own thinking skills, development, was kind of incomplete.*

Anna also said that people did not know what the nursing profession requires. She also thought that the average person did not know how demanding the nursing job is or how many skills nursing work requires, and that she did not like the stereotypical picture of nurses given in the media and on television

*I'm disgusted by the word “Nursey” who's just the typical sort of little blond scrubber that just fools around with the doctors and giggles and puts her cool hand to the feverish patient's forehead wearing a short skirt with her bottom up.*

### 3.5. Life in a New Career: The End of the Story?

In her search for a new career Anna tried to discover one that would better suit her personality as an intellectual, creative, and systematic person. Her decision for changing careers had started as a “sinking” feeling, although the interviews did not reveal a single instance where this intention would have started. This transition process had lasted for many years and was difficult for Anna; indeed a number of times during the interview she remembered that she had often cried and had occasionally felt very tired. However, she presented herself as an active actor who had the power to change a situation that was not satisfactory to her. After the first interview Anna applied to university for a nonnursing discipline, and was accepted. She graduated, and she found a new workplace outside nursing, and also resigned from her permanent nursing job. Anna was the only one of these three nurses who thought that she would never work as a registered nurse again. As Anna shared

*I guess it was pretty much the whole thing and many people have asked me why because this is such a dramatic career change. That it is a bunch of various things.*

According to Betty's story, how she ended up in a different career was as serendipitous as her initial choice to become a nurse. A friend had given her an extra application form for the entrance exam to study nonnursing discipline in the university; after sending in the application she decided to prepare carefully for the exam, studied hard, and was accepted. She managed her financial problems while studying by working as a nurse and by living frugally, which required both self-discipline and sacrificing her own comfort. After graduating from a university, she received a demanding work outside the nursing field. At the time of the second interview Betty had some thoughts of continuing her education in university to doctoral level and also doing some nursing work occasionally under temporary contracts. In Finland reregistration is not required, so Betty would have the possibility to return to nursing.

*At the moment I cannot even imagine what I would be if I had not gone through the nursing school. Although I've thought many times that me being a nurse has been a total waste, that I've been squandering the resources of society or something like that. I think that I would be missing something if I would've not done that. It has given me a lot.*

During the transition process many changes were happening in Cecilia's private life. Her family was very supportive of her career change, because they saw that she was not satisfied with nursing. Cecilia applied to the university to study nonnursing discipline and was accepted. She graduated, and because she was having a university degree, she got a new demanding job in human resource management in a large organization. She enjoyed her new work, even though it was demanding and the workdays were long.

*I do not know if it has been a bad choice, “cause it has made many things possible, but the choice that I've clearly made intentionally has been to leave” cause I've been thinking that it is possible to do things better. But maybe the path that has leaded me away from nursing to here, where I am now, could've been more direct.*

### 3.6. Discussion

In this inquiry, we used a qualitative case-study approach to form an in-depth investigation into the reasons why young registered nurses may leave nursing profession. This study produced three rich, detailed accounts of career transition from a perspective of a young generation. The results of this exploratory qualitative study reflect a shift toward insights into understanding professional turnover as a complex and long-lasting process. These transition processes cannot be simply captured with questionnaires. This could be one reason why earlier quantitative studies do not provide a clear picture of the reasons why nurses leave nursing.

**Figure 1:**
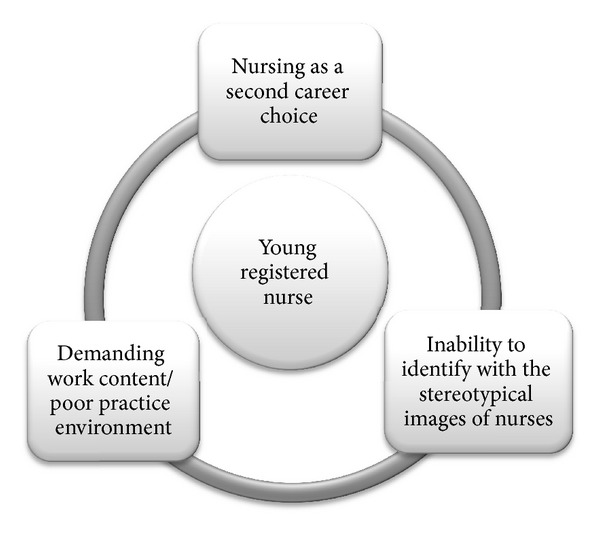
Framework enlightening themes associated with young registered nurse career transition.

All of these career stories are unique but also have similarities (see Figure 1). Firstly, no single incident triggered the decision to leave nursing. None of these young nurses had nursing as a childhood dream; it was more serendipitous that they had ended up as nurses. The work content of nursing was demanding, and the practice environment was not ideal in terms of nurse-patient ratios, rush, shift hours, working contracts, salary, and general appreciation. Furthermore, these young women felt that they did not fit to the stereotypical picture and image of nurses. And finally, nursing was not able to provide the career development possibilities and intellectual challenges that these women were able to gain by applying to university studies and by starting a new career. Although these nurses narrated their career changes as having happened serendipitously, they also constructed themselves as active and competent actors. Throughout their career transition process, these nurses had the possibility to make financial and personal sacrifices for a new education, and they were supported by their families. 

Findings of this inquiry support the work of others; for example, in Takase's [18] literature review turnover intention was described as a multistage process, with a range of factors considered as the antecedents. Additionally, according to Cheung's [63] qualitative interview study of former nurses the decision to leave the profession was a difficult one for nurses, and represents a complex psychological process. The case studies here present three phases of a career transition: nurses reassessed their work; they began the transition process (e.g., by getting a new education); and finally there was a socialization phase for the new job. Trice and Morand [64] described similar phases based on the literature: separation from the old role, transition, and then integration into the new role. As typical for voluntary career transitions these nurses had both time and the possibility to consider options for new education and for a new career [65]. There was no single particular experience or crisis that made these nurses leave the profession. This is contrary to the findings of Cheung [63] and Morrell [66] studies, and where also a single, jarring event or shock initiated nurse's thoughts of quitting. 

As seen in this inquiry, the long-lasting process of career transition can actually start even before young nurses enter the nursing education. None of these nurses who shared their stories held nursing as a childhood dream; they described their career choices as a “second career choice,” or that they had “drifted” into nursing, rather than as a purposefully selected occupation. After being a while in a nursing career all of these nurses applied to their primary educational choice, to study at the university. This finding resonates with previous research. Nursing career chosen as a “default choice” [22] or not having nursing as a childhood career dream [67] has been associated with a shorter tenure in nursing. Also a study conducted in Sweden gave similar results; of graduates (*N* = 672) who were negative or uncertain about their career choice (26%), a majority had considered leaving the profession [68]. In an earlier study by Santamäki et al. [69], approximately one-third of Finnish nurses (35%, *N* = 3,352) reported that they had chosen nursing coincidentally, or as a second-best alternative. By investigating nurses' longitudinal career stories there is an opportunity to gain understanding of the complex dynamics of why nurses choose their career [70] and how leaving considerations and behaviors evolve over time [40]. However, future research is needed into whether the motives for choosing nursing career affect the length of the career in nursing.

According to these young nurses stories, the work content of nursing was demanding, and the nursing practice environment was not ideal in terms of nurse-patient ratios, rush, shift hours, and general appreciation. Nurses felt tired because they could not provide as adequate nursing care as they were willing. Nurses' narratives bought up ethical dilemmas associated with not being able to provide humane and proper care. Not being able to do one's best and not being able to influence working conditions were in contradiction with the talent and ambitions of these young women. Similar themes came up in McGillis Hall and Kiesners [71] study, where nurses related narratives about guilt and over commitment when the working environment prevented them from providing complete and high-quality care. We believe that narrative stories can provide “a window into people's beliefs and experience” [72, page 209] and can provide richer understanding of how young nurses experienced the reality of nursing and how these lived experiences affected their career decisions. 

Young nurses reproduced cultural images and stereotypes of a nurse's role and behaviour. They described what kind of people nurses are supposed to be: nurturing, altruistic, and willing to serve. Similar traditional stereotypes of “good women” have been presented on media and in public when nursing has been presented as a “virtue script” [73]. Nursing, as paradigmatically women's work, has been historically devalued [74]. Nursing profession has been associated with femininity [75] and powerlessness [76, 77], and stereotypical public images—such as angels with pretty faces and empty heads, physicians' handmaids, or naughty nurses—still exist in Western countries [77]. These young nurses did not, however, place themselves within these expected stereotypic images of nurses. This could be one reason why these nurses left profession—talented and bright women did not fit the stereotypes of nurses as nurturing and serving and of being lower in the hospital hierarchy. 

As seen in this inquiry, public stereotypes can have negative impact on nurses' self-esteem [78] and could lead nurses to leave their profession [79]. Although there is considerable amount of research concerning stereotypes and images of nurses [77, 80], a search of the literature revealed only few earlier studies where the low status of the profession was connected to nurse turnover [78, 81]. More quantitative and also qualitative research is needed on how public stereotypes and image of nursing possibly affect young nurse's turnover. Stories can reveal deeply hidden assumptions that influence the actions of people [72]; through stories we can better understand how nurses explain and make sense of the stereotypes and images associated with the profession. By investigating in-depth stories there is a possibility to gain deeper understanding of the historical, sociocultural, and gendered perceptions which partly could lead younger generation of nurses to leave their profession.

In these stories, young nurses described themselves as talented and determined to go ahead in their career, attributes not historically attached to nurses [77, 80]. For example, in the UK [82, page 305], media tend to view “ambition in nurses as being an undesirable characteristic.” Nursing profession was not able to provide the career development possibilities and intellectual challenges that these women were able to gain by applying to university studies and by starting a new career. Similar findings have been discussed in other studies. For example, in a study by Salminen [15], RNs who judged that they had the potential to carry out more challenging tasks had a higher intention to leave the profession. Additionally, Hasselhorn et al. [12] found that former nurses had indicated “too low demands” of nursing work as one reason to leave the profession, and former nurses in a Swedish study by Fochsen et al. [81] stated that a lack of professional opportunities and restricted professional autonomy were central reasons for leaving.

Most of this earlier literature was focused on turnover from the perspectives of organizations or professions, with the researchers viewing it as a preventable or negative result [11, 27]. However, as seen in this inquiry, turnover can also be beneficial for an individual nurse [12, 23]. Changing workplace, and even career, can provide nurses with the opportunity to move to positions better suited to their motives, ambitions, skills, and career goals. According to Hasselhorn et al. [12], a proportion of nurses with an intention to leave the profession are young, highly qualified, and seeking a new challenge. In light of this study, we think that it would be beneficial to health care systems to retain this group of nurses within the profession by opening up to them new possibilities and challenges within nursing. On the contrary, nurses who display a low level of work ability might benefit from leaving the nursing profession. In-depth investigations of career stories could give a broader perspective to the research of nurse's turnover, by focusing on career transition from a perspective of an individual nurse and by enlightening how a single nurse can benefit from a change of career. 

What kind of new knowledge of nurses' professional turnover can we expect to get based on qualitative case-study approach? The purpose of this inquiry was not to seek universal explanations of why young nurses leave nursing profession. The results are not meant to be generalized to a certain population [44] as case study research commonly needs to be generalized theoretically [47, 53]. It is clear that no one methodology by itself will be able to address nurse turnover as a multifaceted phenomenon effectively. There is still a need to statistically measure and model variables connected with turnover [40]; also interventions to reduce turnover intention need to be developed and tested [18]. The advantage of large sample sized research is the breath and the empirical generalizability of the evidence [47]. However, as Flyvbjerg [44] remains, the disadvantage of this kind of knowledge is one of depth; therefore, qualitative case studies are needed as well. 

The findings of this study are significant for three reasons. First, nurses were given an opportunity to tell their own story of why they had initially intended to leave, and why they eventually left, nursing. The voices of nurses who have intended to leave nursing have rarely been studied from their own perspective. Second, in contrast to the majority of previous studies that have mainly focused on turnover intentions and not actual turnover, a unique feature of this study is its longitudinal nature, which allows the reader to follow nurses through the process of their career transition. Third, nurses here provided stories of negative images and stereotypes in relation to their intention to leave nursing; in earlier studies of professional turnover, nurses' own negative stereotypes and images have not been significant factors in causing an intention to leave. Furthermore, it provides unique real-life contextual knowledge of young nurses' experiences of leaving a nursing career. This knowledge can be used as a basis for further studies and in discussions concerning nurses' intentions to leave nursing from a generational perspective. More in-depth research is needed in order to better understand why nurses leave, and even more significantly, what could motive the younger generation of nurses to stay in nursing. In addition, there is also a need for longitudinal research that tracks nurses throughout the career change processes. 

### 3.7. Limitations

The findings should be viewed in light of the study's limitations. The findings reported in this study may be limited due to the sampling methods which were used. It is possible that the nurses who volunteered, and thus were prepared to tell their career stories, were different from other nurses who had also considered leaving the profession. These nurses were interested in the subject under investigation, they had all thought about the motives for why they had an intention to leave and were willing to narrate their experiences. All of the nurses who participated were open to the experience, sociable, and talkative. These are all also characteristics that are associated with persons who are more likely to change their career [21]. 

All of these nurses had the personal and financial opportunities to gain a new education at university, something that is not possible for all nurses with an intention to leave. Furthermore, the participants were all aware of the interviewer's occupation as a registered nurse; thus it is possible that this knowledge may have influenced the way in which they recounted their career stories. They were also aware that the topic of the research was the intention to leave nursing; this in turn might have brought up more negative issues from their career, in order to justify their reasons for leaving it.

## 4. Conclusions

The use of the qualitative case study method made it possible to examine nurses' career turnover as an ongoing and multidimensional process, in which several causes consequently impacted on the final decision to leave the nursing profession. We demonstrated that narrative career interviews are a feasible way of collecting data regarding career transition processes. We also claim that in-depth narrative stories can provide unique real-life contextual knowledge of nurses' experiences when living through these kinds of career transitions. We conclude that the qualitative case-study approach is a potential and novel way of doing turnover research in nursing science. Given the complex nature of nurse turnover, such a qualitative, in-depth approach is essential, alongside with quantitative studies, for a sound development of the nurse turnover research.
